# Supplementary material for: Are carbon emissions trading and green financial instruments synergistic? -Comprehensive quantitative research based on content analysis
Source: PLoS One. 2024 Mar 7;19(3):e0298601. doi: 10.1371/journal.pone.0298601 (PMC10919601; doi:10.1371/journal.pone.0298601)
Supplement: S1 Data — (ZIP) [file pone.0298601.s001.zip › Data/地级市Z_F工作报告.zip!/╣π╢1⁄2/╒┐╜¡╩╨/└┤╫╘╣1⁄2╓┌║┼í╛┬φ┐╦╩2╛▌═°í┐_2003.docx]

各位代表：

　　我代表市人民政府向大会作政府工作报告，请予审议，并请政协委员和其他列席人员提出意见。

2002年工作回顾

　　2002年，市政府在省委、省政府和市委的正确领导下，在市人大及其常委会和市政协的监督支持下，全力推动经济和社会发展，全市国民经济实现较快增长，精神文明建设和社会各项事业取得显著成效。全年完成国内生产总值461.33亿元，增长8.7％。市十届人大五次会议《政府工作报告》提出的经济社会发展主要目标如期实现，重要工作任务基本完成。

　　——工业化进程加快。确立了强化工业主导地位、加快工业化进程的指导思想，作出重要部署，使工业发展活力增强，速度、效益、质量同步提高。工业总产值443.96亿元、增长14.8％，工业增加值145.95亿元、增长12.1％，分别比上年提高3.7和2.9个百分点。规模以上工业经济效益综合指数为163.8％。非公经济增加值219.7亿元，增长14.6%，占国内生产总值比重47.6％。股份制、外资、其他经济类型企业分别增长6.3％、25.9％和13.4％。全社会用电量29.5亿千瓦时，增长12.4%，其中工业用电量增长12.8%，大工业电价降至0.55元/千瓦时。结转和新开工技改项目75个，总投资25.86亿元，其中6个国债项目、18个省重点项目，年内完成工业企业技改投资8.54亿元，增长58.4%。各项改革深入推进，市直３2家劣势企业有序退出市场。调整和完善国企监管运营体系，企业普遍建立法人治理结构。对７家授权经营企业和２家资产经营公司实行经营目标责任制，资产经营公司与权属企业签订年度经营考核责任制。企业全面推行厂务公开制度、大宗材料公开竞价采购制度，激励和规范企业经营行为，保证国有资产安全与增值。制糖企业产权改革加快，糖业生产稳步发展。优势企业的素质和竞争力增强，企业创名牌取得初步成效，获得省名牌产品1个、著名商标5个和国家免检产品2个。

　　——农村经济稳健发展。农业总产值191.1亿元，增长5.9％；农业增加值119.15亿元，增长5.4%。乡镇企业总产值365.62亿元，增长2.2%。农业生产条件改善，投入水利建设资金3.7亿元，重点扩大雷州半岛西南部改水治旱范围，完成治旱工程228宗，新增灌溉面积18万亩，恢复和改善农田灌溉面积65万亩，基本实现治旱预期目标；南亚热带农业示范区办起一批各具特色的示范点。投入1.7亿元新建和改造县乡公路640公里，全市公路密度比上年提高2.6个百分点。结构调整步伐加快，外运菜、水果、蚕桑、花卉等高效作物种植面积有所增长，畜牧业生产比重提高。农海产品流通加工活跃，农产品流通企业发展到1601个，流通产值增长8.5％；开通了“海上绿色通道”，促进农产品多渠道流通。市级以上农业龙头企业销售收入45.22亿元。恒兴集团和丰收糖业公司被评为国家级农业龙头企业。以水产品批发市场为龙头，育苗、养殖、销售、加工等各个环节相配套的对虾产业链基本形成。农业标准化工作取得成效，制定了12个大宗农产品地方质量生产标准，１４个产品通过中国绿色食品认证，23个农产品被评为农业部名优产品；建成5个省级、1个国家级农业标准化示范区和南方国家级林木种苗示范基地。扶贫、减负工作成效明显，解决困难群众子女入学、住房、就医、打官司“四难”问题力度加大，取消１７个涉农收费项目，年减轻农民负担５０００万元。农村电网改造累计投入7.9亿元，农村住宅到户电价降低到0.79元/千瓦时以下，年减轻农民负担1.38亿元。以县(市)为单位实现城乡同网同价，农林场、盐场用电管理体制改革全面完成。扶持本市少数民族奔小康工作取得初步成效，华侨农场体制改革开始启动，乡镇撤并和勘界工作顺利完成，粮食购销市场化改革进展顺利。

　　——外向带动战略有效实施。抓住国家加入世贸组织的有利机遇，积极扩大出口，外贸出口总额5.46亿美元，增长35.7％，其中水产品加工出口9497万美元，增长89%。出口超过１０００万美元的县（市、区）有9个。招商引资势头良好，合同利用外资1.85亿美元、增长44.6%，实际吸收外商直接投资1.1亿美元、增长9%。国内外一批大企业集团在我市投资兴建项目，为经济发展增强了后劲。

　　——第三产业加快发展。第三产业增加值170.6亿元，增长8.5％；社会消费品零售总额205.72亿元，增长8.8％。以创建中国优秀旅游城市为契机，全民参与创优活动，有效推进了城市建设和旅游业发展，“创优”已通过国家旅游局组织的验收。全市接待游客443.27万人次、增长21.6％，旅游总收入29.5亿元、增长21.9％。流通业有新的发展，湛江世贸大厦、爱华百货商业广场和霞山、赤坎商业步行街投入使用，霞山水产品批发市场被评为省大型中高级批发市场。港口货物吞吐量３586万吨，增长26％，其中湛江港完成货物吞吐量２６27.4万吨，增长19.2%。区域经济合作活跃，组团参加多项经贸洽谈活动，成功举办’２００２湛江工业博览会。房地产开发投资10.67亿元，增长28.6％。邮电业务收入18.3亿元，增长10.3％，其中邮政业务收入1.37亿元、增长10.4%，电信业务收入8.03亿元、增长1.1%，移动通信业务收入8.91亿元、增长19.7%。电子政务建设稳步推进，企业信息化逐步推开，电子商务在服务业加快应用。

　　——重点建设项目进展顺利。全社会固定资产投资95.51亿元，增长12.6％。粤海铁路通道湛海线投入运营，南油文昌油田、粤海饲料厂６万吨对虾饲料、华德力公司电力开关柜、华丽金公司ＤＶＤ生产线、米克化能公司６万立方米液氨储罐等项目和110千伏兴隆、金湾输变电工程建成投产，寸金纪念广场等一批市政工程项目相继完成，湛江港３０万吨级油码头和10万吨级航道疏浚工程投入使用，5万吨级国家粮食储备库主体工程基本完成，海湾大桥、15万吨级铁矿石码头和90万立方米奥里油储罐项目动工建设，渝湛高速公路粤境连络线和省道平洋线平乐至洋青段、六遂线廉江至遂城段、出省通道廉江至石角线全面施工，大型木浆项目、东兴炼油厂扩建、奥里油发电厂、茂湛铁路、疏港公路、渝湛高速湛江段、30万吨级航道疏浚、湛江港大型物流库区等一批重点项目前期工作加快进行。

　　——城市规划建设管理力度加大。加强规划工作，开始第五次城市总体规划修编和第一次市域城镇体系规划编制。大力整治市容环境，拆除违章建筑、临时建筑13万平方米，新增绿地面积13万平方米。改造小街巷11条共4.6公里，新装、改造小街巷路灯62公里。出台《土地储备管理暂行办法》，建立土地收购、储备及供应制度，规范土地使用权征用和出让行为。出台《城市规划区内户外广告设置管理规定》，加强对城市广告发布经营管理。市区环境卫生有较大改善。依法对新老污染源进行监督管理，拓展了水环境保护和生态环境保护工作，组织实施南、北桥河环境综合整治，赤坎水质净化厂动工建设，城市环境空气质量保持在良好水平以上。编制《市区生态示范区建设规划》，建立起全市生态环境状况基本数据库。市区地震小区划工作基本完成。湖光岩被批准为国家地质公园。

　　——社会保障制度进一步完善。继续落实“两个确保”，全市9.5万名企业离退休人员养老金社会化发放率达到１００％；进入再就业服务中心的国有企业下岗职工基本生活费得到保障，并基本完成安置，已向失业保险制度并轨，完善失业人员社保关系接续。实施社会保险市区统筹，保障能力逐步提高。完成省下达的社保扩面征收任务，企业养老保险参保人数41.1万人，失业保险参保人数34.1万人，保险费收缴率分别为97.4％和97.6％。完善城镇职工基本医疗保险制度，出台并实行困难群体参加职工基本医疗保险的办法，同时调低退休人员住院医疗保险费自付比例，全市参加医疗保险人数达33.3万人。全市有９.3万人纳入最低生活保障，覆盖率为1.3%。就业和再就业工作取得成效，实现劳动力市场全市信息联网，劳务输出、社区就业服务成为就业新渠道，城镇登记失业率为3.5%。

　　——财政、金融形势平稳。全市地方一般预算财政收入15.48亿元、增长20.2％，一般预算财政支出38.57亿元、增长23.8％。加上中央及省的财政返还、转移支付和专项补助等，财政收支基本平衡。年末金融机构存款余额468.92亿元，增长8.7%，其中城乡居民储蓄存款余额360.34亿元，增长11.9％；贷款余额280.83亿元，增长5.3%。继续积极化解中小金融机构和各类基金会金融风险，加大资产追收力度，维护债权人、债务人合法权益和群众的基本利益。

　　——人民生活水平提高。市区居民人均可支配收入7644元，增长8.9％；农民人均纯收入３５５2元，增长3.2％；在岗职工人均年工资总额10519元，增长10.5%。

　　——社会各项事业全面发展。教育“普九”成果进一步巩固，建立和完善了“以县为主”的农村义务教育管理体制，小学生、初中生巩固率分别达到99.3％和97.2％，高考上线人数居全省前列，撤并小学教学点464个，完成“改薄”学校926所，省财政拨款和广州市援建的180所老区学校改造顺利完成，市区建成59所学校校园网络和覆盖全市单向传输的教育无线网，中小学收费实行“一费制”和财务“零户统管”。继续实施《９８８科技兴湛计划》，累计实施省级以上“火炬”和“星火”计划项目121个，建立了省级工程技术中心3家、农业技术创新中心1家、重点实验室3家和市级工程中心10家，现有24家高新技术企业，高新技术产品产值增长52.8％，民营科技企业163家，民营科技产品产值增长一倍。湛江海洋产业基地成为国家级火炬计划产业基地，一批科普单位被评为国家和省级科普教育基地，科技进步对工业经济增长的贡献率提高到43.2％，科技项目财政资金补助实行公开招投标制度。实施“南粤锦绣”工程和“五个一”工程，雷剧《梁红玉挂帅》获得中国戏剧最高奖项“梅花奖”。广播电视中心开始建设，广播电视光纤网络通到县(市、区)，“村村通广播电视”成绩得到巩固，基层文化宣传阵地得到加强，市区开心广场成为市民喜爱的文化娱乐场所，城乡公共文化设施网络初步形成。市区创建省级卫生城市工作得到巩固。医疗技术水平和应急救治能力提高，“１２０”急救网络建成并投入使用，县级以上医疗机构全部实行药品公开采购招标，降低药品价格，减轻群众购药负担4000多万元。无偿献血工作走在全省前列。农村改水改厕工作加强，改厕普及率达40%。群众性体育活动广泛开展，专业竞技体育水平不断提高，参加第十一届省运会获得金牌总数第六位和总分第八位。继续打好计划生育工作翻身仗，完成省下达的人口与计划生育目标管理责任制任务，人口出生率为12.9‰，人口自然增长率为8.76‰。巩固殡葬改革成效，火化率达到97.4％，完成省下达的任务，对“三道三区”坟墓进行全面清理，市新殡仪馆主体工程完成。统计、审计、物价、粮食、侨务、外事、对台、民族、宗教、科普、地方志修编、档案管理、文物保护、气象、“三防”、残疾人事业等各项工作取得新的成绩。

　　——社会主义精神文明和民主法制建设不断推进。坚持“两手抓，两手都要硬”的方针，进一步健全社会主义精神文明建设的领导体制和工作机制。围绕创建省级文明城市和中国优秀旅游城市的目标，加快文明城市建设步伐。积极开展创建文明村和文明小区活动，农村“四通”、“五改”、“六进村”群众性创建活动蓬勃开展，徐闻县创出建设生态文明村的新鲜经验。公民道德教育、双拥工作、国防动员、人民防空、兵役征集和民兵预备役工作不断加强，军政军民更加团结，继续推进再创“全国双拥模范城”工作。民主法制建设和各级政府依法行政、接受监督的规范性及自觉性有所加强。认真处理人民群众来信、来访，积极做好建议、提案办理工作，共办理人大代表议案、建议184件和政协委员提案159件。加强城乡基层组织和民主政治建设，顺利完成第二届村民委员会换届选举和第一届社区居民委员会民主选举。普法教育、法律服务、劳动教养工作得到加强。整顿和规范市场经济秩序取得阶段性成果。信用建设扎实推进，市场公平与法治环境逐步实现。安全生产形势基本稳定。重视妇女儿童、扶贫救灾工作。继续加强党风廉政建设责任制，纪检监察机关纠正部门和行业不正之风、从源头上预防和治理腐败、查处违法违纪案件以及国家机关工作人员廉洁自律等工作取得成效。深入开展以“打黑除恶”为重点的严打整治斗争，集中整治治安重点地区和突出问题，严厉打击“私彩”赌博活动，大力扫除“黄赌毒”等社会丑恶现象，反走私斗争保持高压态势，深入开展对“法轮功”邪教组织斗争，社会治安形势总体稳定。积极预防并妥善处置群体性事件，依法调处一批山林土地滩涂纠纷，各种社会矛盾逐步化解。

　　——整治发展软环境初见成效。加快政府职能、工作方式和工作作风转变，政务环境进一步改善。在全市开展机关作风测评活动，服务意识增强，办事效率提高。推行办事程序公开化、规范化，建立市政府行政服务中心，为企业和群众提供“一站式”服务。部分县（市、区）和市直窗口部门也设立了服务中心或服务大厅。在乡镇政府(街道办事处)和县级以上政权机关全面推行政务公开和收费公示、办事工作日、办事柜台化、投资代理制等，方便企业和群众办事，逐步树立政府良好形象。

　　各位代表!

　　过去五年，市政府以邓小平理论和“三个代表”重要思想为指导，认真贯彻省委、省政府现场(湛江)办公会精神，按照省委提出的“抓班子，保稳定，促发展”，“发挥湛江优势，推动经济发展尽快进入快车道”的总要求和市委的工作部署，紧紧依靠全市人民，恪尽职守，努力工作，迎难而上，胜利完成了市第十届人民代表大会提出的目标任务，经济社会发展走出低谷并实现重要转机，为湛江在新世纪加快发展打下了坚实基础。2002年全市国内生产总值比1997年增长43.9%，年均增长7.6%。工业化进程加快，一批临海型和资源型工业大项目上马建设，民营经济发展迅速。实施改水治旱工程和建设南亚热带农业示范区，农业和农村经济结构调整取得成效。第三产业发展加快，展开以湛江港“三大工程”为重点的物流基地建设，商贸业、旅游业、服务业活跃兴旺。全力落实外向带动战略，外经贸工作上了新台阶，成功举办了四届经贸博览会，拓展了招商引资新途径和领域。基础设施建设进程加快，一批交通、通信、能源、市政、旅游等重点项目顺利建成。城市规划建设管理力度加大，环境保护取得较好成效，城市功能增强，城市品位有所提升。以糖业改革为突破口的国有企业改革脱困“三年两大目标”基本实现，国有资产监管运营体系逐步建立，各项配套改革进一步深化，社会保障体系框架、制度逐步建立完善。党政机关机构改革顺利完成，软环境整治初见成效，政府自身建设得到加强。人民生活水平有新的提高，城乡居民居住条件不断改善。困难群众“四难”问题解决力度加大，农民负担进一步减轻，就业工作开辟了新的渠道。城乡精神文明创建活动扎实推进，城乡文明程度不断提高。国防建设和军政军民团结成效显著，先后荣获“全国双拥模范城”和“广东省双拥模范城”二连冠称号。教育巩固了“两基”成果，素质教育得到重视和实施，教学质量明显提高，科技贡献率快速提高，被评为全国科技进步先进市。文化、体育事业取得可喜成绩，新闻出版、广播影视事业不断发展。城乡医疗卫生保健网逐步健全，居民健康水平明显提高，获得省级卫生城市称号。计生工作努力打好翻身仗，殡改工作跨入全省先进行列。高度重视依法治市工作，民主法制建设扎实推进。社会治安综合治理工作取得明显成效，社会秩序稳定。

　　五年来，我市两个文明建设的成就来之不易。这些成就是中共湛江市委正确领导和决策的结果，是各级人民代表大会及其常务委员会和人民政协监督支持的结果，是全市人民万众一心、团结奋斗的结果，是中央、省驻湛单位和各方支持配合的结果。在此，我代表市人民政府，向各级人大代表和政协委员，向辛勤工作在各行各业的广大工人、农民、知识分子、干部、驻湛人民解放军、武警部队官兵、公安干警和社会人士，向港、澳、台同胞，海外侨胞和关心支持湛江建设的国际友人，向信任和支持政府工作的全市人民致以崇高敬意和衷心感谢！

　　我们清醒地看到，我市经济社会发展中还存在不少亟待解决的困难和问题。主要是：艰苦创业精神与市场竞争意识不强，开拓创新力度不够；工业主导地位不强，工业化程度较低；县域经济实力薄弱，经济外向化与农业产业化程度较低；国有企业产权改革步伐不快，非公经济放手发展不够；社会经济总量小，人均国内生产总值总量不到全省平均水平的一半，城市居民和农民生活水平较低；财政支出压力大，社会保障能力弱；城镇化水平较低，城市规划建设管理较为滞后；计划生育工作基础不牢；生态环境有待优化；政府职能和机关作风、社会法治环境与新形势、新任务、新发展不尽适应，影响社会稳定的因素不少。湛江要全面建设小康社会,机会与挑战并存，希望与困难同在，任重而道远！我们比以往任何时候更加需要增强忧患意识和竞争意识，增强责任感和使命感，倍加顾全大局，倍加增强团结，倍加维护稳定，在发展中解决存在的困难和问题。

2003年工作安排

　　党的十六大提出全面建设小康社会的奋斗目标，极大地激发起全市人民振兴湛江的强烈愿望。国家加入世贸组织全面扩大开放，实施西部大开发战略继续扩大内需，广东省实施区域协调发展战略加大对粤西地区支持，都将为我市经济社会发展创造极好的发展机遇。发挥资源、区位和港口优势以及当前良好的发展势头，是新一轮加快发展的坚实基础。市第八次党代会为全面建设小康社会制定了奋斗目标，明确了今后的工作方向和任务。特别是胡锦涛总书记最近到了湛江视察，对湛江加快发展寄予厚望。我们必须坚持把发展作为第一要务，坚持从湛江实际出发，工业立市，以港兴市，加速发展，努力赶上全省率先基本实现社会主义现代化进程。

　　今后五年的奋斗目标是：国民经济持续快速健康发展，国内生产总值年均增速高于全国、全省平均水平。构建起以临海工业和农海产品加工业为主要支撑、三大产业协调发展的产业结构，经济运行质量和效益持续提高，工业化、农业产业化、城镇化、外向化水平显著提升。社会主义市场经济体制进一步完善，经济运行机制充满活力。人口、资源、环境协调发展，基础设施、生态环境和人居环境明显改善，成为国内最适宜创业发展和生活居住的城市之一。精神文明建设和政治文明建设进一步加强，文明法治程度提高，科技、教育、文化、卫生、体育等各项社会事业全面进步，人民生活水平明显提高。通过五年的艰苦奋斗，实现全面建设小康社会的第一步目标，初步建成现代化新兴港口工业城市。

　　今年政府工作总的指导思想是:以邓小平理论和“三个代表”重要思想为指导，全面贯彻党的十六大、省第九次党代会和市第八次党代会精神，坚持强化工业主导地位，推进农业产业化经营，实施外向带动战略，增强港口带动功能。切实加强精神文明建设和民主法制建设。依法行政，从严治政，继续转变政府职能，改进工作作风。统一思想，振奋精神，抢抓机遇，开拓创新，切实加快经济发展和社会全面进步，为全面建设小康社会扎实起步。

　　今年经济社会发展主要预期目标是：国内生产总值增长9％，其中第一产业增长5％，第二产业增长11％（其中工业增长１２％），第三产业增长10％。固定资产投资增长１２％，社会消费品零售总额增长１０％，外贸出口增长１０％，实际利用外资增长１０％，地方财政一般预算收入增长10％，居民消费价格指数持平或略有上升，城镇登记失业率控制在3.5％左右，人口自然增长率控制在9.2‰以下，城镇居民人均可支配收入增长5％，农村居民人均纯收入增长4％。

　　今年的主要任务和工作重点是：

　　一、切实加快工业化进程，努力壮大经济总量

　　按新型工业化要求加快结构调整。重点发展临海型工业和资源型加工业，培育大批企业成为法人实体和市场竞争主体，扶持一批有发展潜力的大型企业集团，提高企业的综合竞争力。以信息化带动工业化，加快利用高新技术和先进适用技术改造提升机械、家电、化工、建材、饲料、纺织、食品、造纸、包装等传统产业，使其做大做强，重现竞争活力。加快中小企业技术改造，增强市场开拓能力和发展后劲。推进生产、管理和产品标准化工作，鼓励企业创名牌，对获得国家级和省级名牌产品、著名商标、驰名商标的企业给予专项奖励。每年公布全市企业50强，评选10佳工业企业。

　　加快大中型工业项目建设进程。充分利用海洋、港口和南亚热带资源优势，吸引国内外大企业来我市投资工业项目，全力支持中央、省属企业在我市发展壮大。支持中石化东兴炼油厂５００万吨炼油项目扩建，支持中石油等三方合作的１２０万千瓦奥里油发电厂、大中纺织公司2.5万锭纺纱和华农集团二、三期及富虹集团100万吨大豆加工等项目建设，继续做好大型木浆项目的前期工作。成立重点项目办公室，统筹协调和跟踪落实大中型项目。

　　建设好经济技术开发区和工业园区。加快开发区和试验区的建设步伐，根据规划和发展需要，调整土地使用功能，降低土地价格，吸引工业项目。启动建设开发区延伸区前期工作，使开发区、试验区成为对外招商引资和发展工业的重要载体。市区要集中一定财力，加快滨海、官渡、麻章三个工业园的建设步伐。合理规划，分步实施，建设以临海型石化工业、家用电器工业、海产品加工工业和饲料工业为主的各类专业性工业园区。鼓励有实力的企业特别是民营企业投资兴办工业小区。

　　发展壮大县域工业。把发展工业特别是农海产品加工业作为县域经济结构调整的主要任务。各县(市、区)都应兴办工业园区，镇镇都要兴办工业企业，下大力气抓好招商引资工作，千方百计引进工业项目，提高工业在经济总量中的比重。支持乡镇企业改革发展，引入多元投资主体，盘活资产存量。推进农海产品标准、商标、包装、保鲜、出口、流通工作。建立对县(市、区)政府发展工业的考核奖励制度。成立中小企业局，做好县(市、区)、镇两级项目引进和兴办企业的协调与服务。

　　全力推动民营经济发展。树立民营经济是社会主义市场经济重要组成部分的观念，对民营企业实行“政治平等、政策公平、法律保障、放手发展”的方针，对民营经济不限发展比例、不限发展速度、不限经营方式、不限经营规模，放胆、放手、放开发展，努力为民营企业创业人员营造社会上有地位、政治上有荣誉、经济上有实惠的良好发展氛围。放宽市场主体准入条件，凡符合国家法律法规、产业政策规定以及加入世贸组织后对内对外开放的行业和投资领域，都对民营企业开放，依法保护民营企业合法财产和权益。采取有效措施激活民资，鼓励、吸引在外投资的本地企业家回乡办企业。创造公平竞争环境，鼓励和支持民营企业参与经济结构调整，开发新产品，努力创名牌。鼓励、引导民营企业加快由家族式向现代企业制度转变，实现专业化生产和现代化管理，不断提高市场竞争力，把企业做强做大。每年评选优秀民营企业和优秀民营企业家。

　　继续深化国有企业改革。改革和完善国资监管运营体制，实行权利、义务和责任相统一，管资产和管人、管事相结合，建立规范的现代企业制度和法人治理结构。按经营业绩调整企业分类，完全取消企业行政级别，把企业彻底推向市场。两家资产经营公司要加大资产运营力度，加快盘活存量资产，将优质资产集中于优势企业。授权经营企业既要自身发展壮大，又要发挥带动整合作用。大力推进产权制度改革，放开国资股权比例，促进投资主体多元化，上半年把市属若干家国有企业在市产权交易中心挂牌招商。加快糖厂产权改革步伐，发展订单糖业，办好糖业龙头企业。继续以多种形式放开搞活中小企业，依法关闭破产劣势企业。完善产权市场交易制度，规范产权交易行为，防止企业转制过程中国有资产流失。完善国有资产保值增值考核办法，继续实行经营责任制，强化利益激励和制度约束机制。加快改革企业分配制度，对企业经营者和技术骨干试行年薪制、期权制等激励办法，鼓励和支持技术、管理等生产要素参与分配。推广国有企业经营人员公开招聘和选拔。减轻企业负担和历史包袱，分离企业办社会职能，按政策分流安置富余人员。完成华侨农场体制改革。

　　二、推进农业产业化经营，实现农业增效农民增收

　　构建南亚热带农产品基地。充分利用南亚热带气候、土地资源和良好的农业基础，引导千家万户农民闯市场，按照市场需求调整农业经济结构，改变粗放落后的生产方式。着力引进和培育众多的农业企业，形成农业开发的集约力量，加快建设造纸、制糖、对虾、珍珠、果菜、畜牧等6大产业链和一批农业示范基地，提高农业效益，增加农民收入。

　　抓好良种的培育、引进和推广。大力推广良种良法，推进农产品优质化、区域化，提高农牧业产量、质量和价值。积极做好本地优良品种的选育推广，巩固发展红橙、荔枝、芒果、菠萝、香蕉等主导产品，大力发展南亚热带特色的水果、蔬菜、花卉、蚕桑、剑麻、速生丰产林等经济作物和对虾、珍珠、鲍鱼、湛江鸡等养殖品牌，积极引进和推广外地优良新品种，形成新的优势产品。依法规范种子经营行为。健全农业技术推广网络，鼓励科技人员以技术入股。

　　发展海洋捕捞、养殖和加工。积极发展现代化的捕捞船队，抓好中深海捕捞和远洋渔业。加强渔港建设，做好硇洲国家中心渔港报批建设。加快人工渔礁建设，营造海洋渔业牧场。依法维护、改善养殖和生态环境，抓好标准化生态养殖。大力发展水产品加工、保鲜、出口，提高水产品的质量和效益，支持建设国家863计划海水养殖种子工程南方基地，努力把我市建设成为南方最大的对虾、珍珠和罗非鱼养殖加工交易出口区域性中心。继续做好北部湾捕捞渔民转产转业工作。

　　大力培育龙头企业和种养大户。稳定和完善农村土地承包关系，建立农民“自愿、依法、有偿”的土地使用权流转机制。积极发展农村专业合作经济组织，大力推广“公司＋农户”等方式，发展农业产业化经营。集中力量扶持各级农业龙头企业，增强其发展后劲和带动能力。支持恒兴集团、丰收糖业公司、畜牧业联合公司、东方剑麻集团等国家级和省级农业龙头企业发展壮大；支持中鑫公司增强省级龙头流通企业辐射能力，并做好新水产品批发市场筹建工作，使该市场成为区域性海产品流通基地。加大农业项目招商引资力度，吸引外地农业投资者进入农业开发和农产品加工领域，发展外向型农业。

　　加大农业基础设施投入。围绕节水灌溉、人畜饮水、乡村道路、江海堤围、农村水电等工程，扩大投资规模，加快建设进程。继续抓好扩库硬渠节水治旱规划项目及水、电、路、机井等配套项目。加大大中型水库、江海堤围达标和大型灌区节水改造工程建设力度，搞好小型水库、水闸除险加固。继续抓紧实施解决群众饮水困难工程，力争用两年时间解决全市60万群众饮水特别困难问题。多渠道、多形式增加农业投入，鼓励个人和社会资金投入生态农业和农业基础设施建设。增加对农业科研、农科普及、技术开发、病虫害防治等方面的投入，提高农业生产效率和防灾减灾能力。继续加强农业机械化建设。

　　健全农业社会化服务体系。加强教育培训，组织学习各种实用技术，提高广大农民素质。继续抓好农业标准化生产，加快采用国际农业标准，实施农产品质量安全标准，建立健全农产品质量监督检测体系。组织市级优质农产品评选，培育名牌商标，打造湛江农海产品品牌。抓好农产品种植、加工、贮藏、保鲜、包装和果树高位嫁接、无公害生产等技术推广。加强动植物防疫网络建设，推行动物免疫标识制度。积极发展农产品行业协会和农民专业合作组织，加强行业自律。建立、完善农业信息网，使信息网络加快向乡镇、农业龙头企业、批发市场、中介组织、经营大户延伸扩展。加强农村市场建设，引入现代流通方式，增强我市农产品市场销售能力。设立市果菜办公室，加强对全市果菜加工与销售的引导、协调与服务工作。

　　加快城镇化进程。重视对小城镇作出科学合理的规划，严格按规划进行建设。把发展乡镇企业同加快城镇化进程结合起来，引导鼓励乡镇企业向小城镇集聚，提高小城镇的市场化程度和规模效应，带动二、三产业发展。改变城乡二元结构，深化户籍制度、就业制度和福利保障制度三位一体的改革，放宽农民入城镇条件，加快农村劳动力向非农产业转移。依法保护土地资源和生态环境。重点扶持省确定的19个中心镇搞好规划，积极发展工业，做好镇区净化绿化。

　　努力增加农民收入。认真贯彻“多予、少取、放活”的方针，采取多种途径、多项措施增加农民收入，使农民得到实惠。全面实行农村税费改革，按照部署，取消农业特产税，切实减轻农民负担。加大对涉农方面“三乱”行为的专项治理力度，保护农民合法权益。加大扶贫力度，认真解决老边穷地区、水库移民等贫困人口的生产生活困难。继续开展“百家扶百村”、“千干扶千户”一帮一扶贫活动。积极筹集资金，帮助无办公场所的村委会建好办公楼。指导帮助村委会发展集体经济，搞好劳务输出，积极发动引导、组织农村富余劳动力到城镇特别是到珠三角发达地区务工，增加农民的非农收入。

　　三、加快港口和城乡基础设施建设，强化区域性中心城市功能

　　努力增强港口地位。全力以赴加快港口建设，千方百计提高港口吞吐量，想方设法扩大港口辐射面，以开放和竞争的姿态增强湛江作为大西南重要出海通道的作用和地位。近期着力抓好湛江港１５万吨级铁矿石码头改造、３０万吨级航道疏浚、３０万吨级油码头配套等重大工程的建设。改革港口管理体制，实行政企分开，组建港口管理局和湛江港企业集团，由政府接收湛江港所办学校、医院等社会职能，增强港口的竞争能力。抓紧做好东海岛深水岸线开发规划和保护，建设东海岛5000吨级起步码头，续建霞海、流沙港，新建东头山、东参、特呈等3个陆岛码头。

　　以港口为依托大力发展现代物流业。按照“前港后厂、前港后仓、前港后场”的模式，开拓运输、仓储和加工、贸易业务，加快港区物流园区建设，加快建立石油化工、矿砂、钢材、粮食、果菜、海产品等物流基地。办好保税仓，积极申办出口加工区和保税区。大力发展城市商业和服务业，建成霞山水产交易中心市场和鑫海商业广场，加快建设湛江车城二期、徐闻农副产品批发市场二期工程，新建或改造市区一批专业及集贸市场，推动社区商贸超市和连锁便利店建设，改善市区居民的购物环境。继续发展金融、保险等第三产业，为现代物流业做好服务。

　　加快道路桥梁和市政电力设施建设。积极做好各方面工作，切实加快全市交通重大项目建设。公路建设投资约10亿元，其中建设(改造)国、省道123公里，主要包括疏港公路、海湾大桥、渝湛高速高桥至遂溪段、207国道良垌段、省道遂六线廉江至遂溪段、省道砂土路改柏油路42公里。建设县乡公路520公里，改造危桥18座，建设县(区)汽车站场及火车客货运站场8个。市政设施建设投资5.2亿元，安排51个项目，主要是新建(改造)东新路、寸金路、寸金路与铁路立交、广湛路出入口段、椹川大道、机场路、南北桥河交汇口、观海长廊延伸段等工程。电力设施投资3.74亿元，重点新建110千伏麻章、平乐、青平、唐家输变电工程，续建220千伏吴川和110千伏南调、樟铺、北门输变电工程，开展开发区220千伏输变电工程前期工作。邮政通信设施投资6.5亿元，主要进行GSM网络改造和CDMA网络、IC卡电话、小灵通扩容。

　　加快旅游基础设施建设。巩固“创优”成果，加大旅游业发展力度，继续把旅游业做大做强。深化旅游体制改革，鼓励多种成份企业参与旅游业发展，培育多元化的旅游市场主体。继续搞好旅游设施和景区、景点的精品建设。规划建设市区旅游美食街，引入外资建设东海岛高尔夫球场，扶持中国大陆最南端徐闻旅游项目建设。加快旅游景区(点)、旅游饭店的评级评星工作，力争湖光岩风景区和东海岛旅游区被评为国家4A级旅游区以及南亚热带植物园、雷州乌石天成台旅游渡假区评为国家3A级旅游区，协助做好皇冠假日酒店五星级评定。加大旅游市场的开发促销力度，搞好旅游线路的包装推介和旅游纪念品的开发。

　　加快国民经济信息化进程。大力促进企业信息化，推进信息技术在企业经营和产品开发的应用。抓紧与电子商务配套的认证体系、网上支持体系建设。推进电子政务，提高办公自动化程度，办好社会保障系统网络、宏观经济信息网和统计信息网，加快市、县、镇三级信息联网。

　　四、努力扩大对外开放，积极参与国际经济合作与竞争

　　加大招商引资力度。大力开展招商引资活动，市和各县(市、区)都要组织专门的招商引资工作班子，经常性地到海外、港澳台、珠三角、长三角地区招商引资。充分利用港口优势和三大石油公司进入我市投资的良好机遇，继续引进重大石化工业项目，利用好开发区、试验区、工业园区优惠政策和原有国有企业基础如机械制造、电子电器、食品加工等设施及品牌资源、南亚热带农业资源、海洋资源优势及市政道路、港口等基础设施项目对外开展招商引资。强化企业招商引资主体功能，充分调动企业和社会各界招商引资积极性，引导以资引资、以商引资、以项目引资。举办’2003湛江工业博览会，开展较大规模的集中招商引资活动。实行县（市、区）政府招商引资责任制，各县（市、区）近年实际吸收外资要尽快达到1000万美元，每年有较大幅度增长。开发区、试验区要强化外经贸发展排头兵的意识和行为，加大外资项目引进力度，营造投资热点。增强外事、侨务、公安出入境工作服务功能，积极发挥海外侨胞、港澳台同胞和国际友人的作用，促进经济、文化交流与合作。

　　努力扩大外贸出口。开拓多元化国际市场，巩固香港、美国和日本市场，开拓新兴市场，进一步拓展远洋贸易。调整和优化出口商品结构，扩大机电产品、水产品、纺织品、畜产品等本地大宗产品出口，力争培育一批出口名牌和著名商标。继续扶持年出口额超１０００万美元以上企业，着力培育和发展出口额5０００万美元以上的大型外经贸企业集团。支持民营企业开展外经贸业务，帮助民营企业取得进出口经营权和各类出口企业取得各种管理及质量认证。各县(市、区)近年外贸出口要尽快达到2000万美元，保持快速增长。落实各级鼓励外贸出口的政策措施，进一步建立和改善“大通关”的环境。深化外贸体制改革，搞活国有外贸企业经营。继续做好“入世”应对工作，完善对外贸易预警机制。

　　加强地区间经济合作。加强与港澳台地区的经济合作，优势互补，共同发展。发挥自身比较优势，创造条件接受珠三角地区的经济辐射和产业转移。加强与西南各省（市）经贸联系，积极代理西南地区产品进出口，参加大西南各省（市）举办的经贸活动。组织参加粤台经济技术交流会，加大台资引进工作力度。

　　五、加强城市规划建设管理，塑造文明城市形象

　　增强规划的法规性和权威性。规划是城市的灵魂，决定城市发展的水平、质量和可持续性。各级政府尤其是规划管理部门要本着对人民根本利益、对湛江长远发展高度负责的态度，依法秉公办事，维护规划的权威性，任何个人都无权随意改动已经法定程序确定的城市规划。围绕建设现代化新兴港口工业城市、区域性中心城市的目标和加快城市化进程的要求，突出“南方港城、南国风光”特色，以科学精神和超前思维认真做好湛江市第五次城市总体规划修编、第一次市域城镇体系规划编制和七个规划专题研究工作。贯彻“企业进园、房产进区、摆卖进店”的原则，先规划后用地，对未列入城市近期建设规划、没有编制控制性详细规划的地块，一律不得出让。要重视搞好文化、体育设施和海岸景观的规划，多建设绿化公园和文化娱乐广场，合理规划建设停车场和肉菜市场。加强规划对房地产的调控力度，全面控制零散的房产开发，所有房产开发要严格执行国家有关部门规定的建筑密度、容积率、绿地率等重要指标。加大拆除违章建筑、临时建筑和因规划建设需要拆除建筑物的力度，坚决杜绝新的违章建筑出现，全面控制临时建筑，依法追究造成违章建筑和以罚款补办手续的审批者的行政责任。高层建筑、重要地段建筑和小区开发项目，均需提交市规划与国土管理委员会审批。重视做好准备开发项目的修建性详细规划。对风景区及旅游重点单位，更要加强规划审批，严格保护资源。加快旧城改造步伐，启动改造霞山洪屋街、汉口路、东堤路片和赤坎南华市场片旧城区工作。尽快实施城市建设工程规划设计公示制度。改革勘察、设计体制，城区建筑物强制推行设计招投标及方案选优制度。重视无障碍设施建设。

　　多渠道筹集城建资金。用经营城市理念指导城建融资工作，充分发挥城市融资潜力。财政合理安排市政建设重点项目投资，积极争取中长期国债、国家政策和金融机构支持。规范城市建设和管理各项收费的减免，广告经营权等城市公共资源统一规划、公开拍卖，收入所得用作城市建设资金。全面放开供水、供气、环卫、污水和垃圾处理等市政公用设施的建设投资权和经营权，实行特许经营权制度，向社会公开招标选择投资和经营主体。改革公用事业管理体制，园林绿化、环境卫生等单位要加快改造成为企业，鼓励成立民营绿化和清洁公司。定期公布城市基础设施项目对外招商融资，吸引民资和外资进入城市建设领域。简化房改房上市手续，盘活房地产二手市场，促进住房消费，推进一批环境优美的花园式住宅小区建设。

　　切实加强城市管理。全面开展创建“国家卫生城市”活动。修订《市容环境卫生管理办法》，实现市容环境卫生经常化和制度化管理，认真落实“门前三包”责任制。全面治理城市垃圾，扩建冯村垃圾处理场，提高上门收垃圾比例，建设分布比较合理的垃圾转运站，增加垃圾处理设备。巩固市区灭蚊、灭蝇成果，年内实现灭蚊达标。推行土地收购储备和供应制度，政府垄断土地一级市场，实行统一征用、收购、储备和出让，所有用地单位都必须从政府土地管理机构中取得用地。今年开始按用地总需求规模和城市建设规划发展重点，确定土地供应总量，定向定性向社会供地。商业、房地产等经营性用地全部采用公开招标拍卖方式进行土地使用权交易；行政办公、市政建设和文教卫体等用地，经政府集体研究后以划拨方式供地，工业用地价格实行优惠或按成本价供应。加大对城市闲置土地的回收处理力度。严格控制市区和县（市）城区私人建房和风景区内建房，加快城中村改造。加强市区交通秩序管理，调整和增开市区公共汽车线路，取缔摩托车、三轮车和残疾人车非法营运，加快限制摩托车在市区内行驶的工作。取缔影响市容的路边饮食档。保护海岸线，严格控制市区岸线填海，逐年逐段建设霞赤两地相连的观海长廊。组建城市管理行政执法局，相对集中行政处罚权，解决多头多层执法和以罚代管、重罚轻管的问题。

　　做好“城市绿化年”工作。全面开展创建“国家园林城市”活动。组织编制城区绿化规划。认真组织实施“城市绿化年”的各项计划任务，确保新增绿地１００万平方米。规划建设好城市的环城绿带、林荫大道、森林公园和街区绿景，争取人均绿地面积尽快达到８－１０平方米。凡是设计达不到绿化标准的新建项目，各职能部门一律不予批准。规划建设海湾大桥两岸共1000亩的桥头公园、文保1000亩的绿化公园和市区红树林岸带。市区新建10个小公园、小游园，主干道路进行绿化改造。开展园林绿化进街道和单位庭院、小街小巷、生活小区的绿化达标活动。年内建成300公里国、省道“绿色长廊”，三岭山森林公园申报省级森林公园，改造青少年公园和花圃。将各单位的临街围墙拆除或改为通透式围墙，增加城市绿化空间。各城区政府要加大整治小街小巷的力度，搞好各镇政府所在地及国、省道沿线两旁环境的净化、绿化，年内城镇环境卫生要有根本性的好转。

　　六、推进科教兴湛工作，促进社会可持续发展

　　完善科技创新体系。继续开展创建全国科技进步先进示范市和先进示范县(市、区)活动。深化科技体制改革，建立完善以企业为主体的技术创新体系和以大学、科研机构为主体的知识创新体系。继续在规模以上工业企业中建立工程中心和技术开发机构，在农业龙头企业中建立省级农业技术创新中心，争取建立省级以上技术检测机构和重点实验室。促进科技型中小企业发展壮大。建设好国家级火炬计划湛江海洋产业基地和省级农业科技园、农业科技示范基地，筹建省级民营科技园和省级高新技术产业开发区。继续公开招标科技项目，提高财政支持的有效性。加强科普工作，继续建立和完善科普教育基地。发挥科技中介服务机构作用，培育技术市场。设立风险投资专项资金，用于支持高科技人才到湛江创业。完善推进科技进步的人才、投入、成果转化的评估机制和知识产权保护体系。

　　全面推进素质教育。教育要深化改革，优化结构，提高质量，增加投入。继续完善农村义务教育“以县为主”的管理体制，落实“三保”，撤销镇级教办机构。巩固提高普及九年义务教育，坚持中小学“一费制”和免收农村困难家庭子女义务教育阶段书杂费制度，切实解决困难家庭子女入学难问题。加快解决市区和县(市)城区中小学学位不足问题，推进中小学布局调整。优化教育资源，搬迁重建市二小和市二中，扩大湛江一中办学规模，力争湛江一中通过国家级示范性高中验收，做好县(市)示范性高中规划、建设工作。用好省政府老区学校改造专项资金，建好100所老区学校，做好农村薄弱学校改造扫尾工作。全面推进中小学素质教育，加快课程改革和信息技术教育、英语教育进程。以就业市场为导向，整合中等职业教育资源，提高职业技术教育的适应能力。帮助高校解决实际困难，支持驻湛高校做大做强。完善继续教育制度，加强教师队伍培训。

　　积极培养和引进人才。营造聚集人才和人才成长的良好环境。重点培养和引进具有现代科技知识，懂经营管理、懂法律、懂外语、懂计算机和信息技术、懂世贸知识和国际经济事务及国际规则的本科学历以上专业人才，并为他们投身湛江建设提供良好的工作和生活条件。完善利益激励机制，推行技术入股参与分配等政策，落实对有突出贡献的科技人员进行奖励的规定。继续做好培训工作，加快培养适应我市中长期建设需要的各方面人才。

　　十分重视环境保护。进一步落实各级政府环保目标责任制，开展创建国家环保模范城市活动，大力治理污染源，要以保护饮用水源水质为重点，落实水污染整治计划，坚决搬迁或关闭影响饮用水源安全的污染源。加快建设赤坎、霞山和各县级市污水处理厂，实现我市城区生活污水处理零的突破。按照国务院的要求，开征污水处理费。严格控制大气污染物的排放，推进工业污染源全面达标，切实治理污染严重企业，依法关闭污染严重或国家明令禁止的污染企业，燃煤燃油发电厂限期配套脱硫设施。加快城市固体废弃物减量化、无害化、资源化处理，加强对生产生活噪声监管。提高市区机动车尾气达标率，减少空气污染。霞山、赤坎两区建筑工地全面控制现场搅拌混凝土，增加搅拌混凝土市场供应能力。

　　加强生态建设和资源管理。巩固发展造林绿化成果，提高森林覆盖率，保护好市区海湾沿岸和三岭山森林公园，加快市区寸金桥公园等公园湖水的治理。广泛发动农村群众积极改水改厕和美化环境，推进生态示范镇、村创建工作。依法保护和合理开发各种自然资源，建立健全国有资源有偿使用制度，坚持土地、林地用途管制。运用耕地易地开发政策，规划建设4600多亩湛江市交椅岭生态园。严格控制非农用地，保护海洋环境和红树林、珊瑚礁等生态系统。健全环境监测体系，加强地质环境保护和气象预测预报。着力搞好防震减灾和重大工程地震安全性评估工作。

　　巩固计划生育和殡改工作基础。认真执行《人口与计划生育法》和《广东省人口与计划生育条例》，重心下移，强化基础，落实目标管理责任制。抓住依法管理、优质服务两个重点，推进村（居）民自治，下大力气解决计生工作存在虚报瞒报漏报的问题。巩固殡改成果，搞好“三道三区”坟墓清理的扫尾工作，提高全市火化率。

　　七、加强政治文明和精神文明建设，发展社会各项事业

　　加强社会主义民主法制建设。按照建设社会主义政治文明的要求，进一步完善民主决策、依法决策、科学决策机制，认真执行人民代表大会及其常务委员会的决议、决定，认真办理人大议案、建议和政协提案。坚持政府向人大及其常委会报告工作，向人民政协通报情况的制度。发挥民主党派、工商联、无党派人士参政议政作用和工会、共青团、妇联组织的桥梁纽带作用，多方听取人民群众意见和要求。各级政府必须自觉接受人民的监督，建立健全各项监督制度，确保权力真正用于为人民谋利益。健全基层自治组织，完善村民自治和居民自治，完善村务公开和厂务公开，完善职工代表大会和企事业单位民主管理制度。坚持依法行政，从严治政，规范行政权力，严格行政责任，强化行政执法监督，确保政令畅通。抓好“四五”普法教育，加强法律援助，成立公职律师事务所，认真做好行政复议和行政应诉工作。进一步清理不适应新形势、新任务要求的地方规范性文件和其他政策措施。不断改善执法环境，完善执法保障机制。

　　发展先进文化事业。深入宣传学习贯彻党的十六大精神，积极跟上建设广东文化大省的步伐，全面启动创建“省文明城市”活动，对照省文明城市的条件要求，广泛宣传教育，着力提高市民的文明素质。继续整顿文化市场，加强对电子游戏机室、音像市场、网吧等场所的管理。深化文化体制改革，发展文化产业。积极开展社区文化等群众文化活动，推动先进文化“进村入户”。继续实施“文化精品”战略，创作优秀文艺作品，建设富有特色的地方文化品牌，对获得国家级荣誉的有功者实行奖励。合理配置文化资源，加强公共图书馆网络建设。发展广播电视、出版事业，加快广播电视中心、档案馆建设，筹建文化艺术中心。抓好历史文化遗产保护、新地方志编修工作。建设新型社区，优化社区服务，做好城市管理基础性工作。

　　发展医疗卫生事业。深化城镇医疗卫生体制改革，实施区域卫生规划，建立健全卫生监督执法和疾病预防控制体系。切实加强初级卫生保健和农村卫生工作，逐步建立农村卫生服务新体系。做好专科医院发展规划，筹建湛江市传染病医院等专科医院。整顿规范药品市场，规范和扩大药品集中招标采购，有效遏制医疗费用的不合理因素，减轻群众医药负担。积极稳妥开展社区卫生服务。积极扶持中医药事业发展。

　　建设体育强市。发展群众体育事业，构建亲民、便民、利民的体育服务体系，推动全民健身活动广泛开展，突出重点发展竞技体育。扩大现有体育场馆和学校体育场地并向社会开放，推动体育向产业化、社会化发展。各方支持配合，努力承办好省第六届大学生运动会。

　　高度重视维护社会稳定工作。落实社会治安综合治理目标管理责任制等制度，为群众创造安居乐业的社会环境。严厉打击各种暴力犯罪、带黑社会性质的团伙犯罪、毒品犯罪、走私犯罪以及抢劫、盗窃等多发性犯罪，集中整治治安混乱的重点地区和突出问题，积极构建打、防、控一体化的治安工作体系，完善群防群治网络。加强劳动教养工作和对刑释解教人员安置帮教工作，控制重新犯罪率。严厉打击“私彩”、传销、盗版等违法犯罪活动。认真做好国家安全工作。严密防范、坚决打击敌对势力和“法轮功”邪教组织的破坏活动，确保社会政治稳定。

　　依法调处各种社会矛盾。坚持治标与治本一起抓、治理与防范并重，积极主动化解各种社会矛盾，认真负责地处理群众来信来访。加强基层人民调解工作，及时解决实际问题。认真调处山林土地滩涂纠纷等社会矛盾，果断处置突发性群体事件。加强城乡基层政权和基层组织建设，疏通城乡居民民主参与渠道，增强社会管治能力。

　　切实加强安全生产工作。全面贯彻《安全生产法》和《消防法》，进一步落实安全生产责任制和重、特大安全事故行政责任追究制度，推行注册安全员制度。突出抓好取缔“私炮”等安全生产专项整治，加快城市公共消防基础设施建设，加大火灾隐患整改力度，努力减少伤亡事故和重大安全生产事故发生。

　　建立规范的市场经济秩序。完善对市场主体资格和市场行为等方面管理的政府规章。规范有形建筑、土地交易和产权交易市场运作机制，加强对建设工程、经营性土地使用权和国有产权的招投标拍卖行为的监管。规范中介机构活动，建立公平与诚实的机制。严厉打击制售假冒伪劣商品行为，保障人民群众切身利益和生命安全。依法规范市场准入和竞争行为，努力维护生产者和消费者的合法权益。依法加强市场物价监管，防止无序与恶性竞争。切实加强政府、企业和社会信用建设，维护本地区良好信用形象。

　　认真做好社会各项工作。认真实施妇女儿童发展规划纲要，切实维护妇女儿童合法权益，尤其是保障妇女参政议政、劳动就业等权利。提高妇女儿童保健水平，重视儿童培养，建立完善生育保险制度。关心支持残疾人事业，推进残疾人康复和就业。全社会重视解决老龄人口社会保障和精神文化生活问题。增强全民国防观念，增进军政军民团结。重视和支持国防建设，认真做好国防动员、武装、民兵、预备役、人民防空工作，努力争创全国双拥模范城“二连冠”。进一步做好侨务、外事、民族、宗教、粮食、物价、统计、城调、气象等工作，推动社会各项事业全面进步。

　　八、做好就业和社会保障工作，提高人民生活水平

　　改善就业环境和增加就业岗位。落实政策措施，建立积极就业政策的基本框架和以培训促就业的新机制。拓宽就业渠道，大力发展社区服务业、非公有制经济、劳动密集型企业，促进就业新观念和市场机制形成，鼓励劳动者通过灵活多样形式实现就业。建立就业援助制度，为就业难的国有企业下岗失业人员提供免费培训和岗位援助，力争年内新增城镇就业岗位3万个。完善就业服务体系，健全劳动力市场网络，增设公益性职业介绍机构，为下岗失业人员提供职业介绍、就业指导、职业培训等“一站式”免费服务。在社区建立劳动保障工作场所，为下岗失业人员服务。举办公益性大型劳务招聘会和下岗失业人员、军嫂等专场招聘会，在珠三角地区建立我市劳务输出服务站，切实做好劳务输出工作，力争年内劳务输出12万人。依法保护包括外来务工者在内的全体劳动者合法权益。

　　健全社会保障体系。完善城镇职工养老、失业、医疗保险和城乡居民最低生活保障制度。依法扩大社保覆盖面，强化社保基金征收，确保失业人员保险金和企业离退休人员养老金按时足额发放。各有关部门要工作细致，掌握实情，将符合条件的城乡居民纳入最低生活保障范围，做到应保尽保。建立对困难群众的援助机制，完善包括医疗卫生、社会福利、教育、司法援助、慈善救助等多方面的社会援助网络，使困难群众及时得到帮助。调整财政支出结构，增强政府实施低保制度和养老等制度的保障能力。处理好医、患、保关系，建立协调机制，加强对社保定点医院和零售药店的管理。铺开农村合作医疗保障制度工作，力争今年全市农村参加合作医疗人数达30%以上。积极发展商业保险。推进企业退休人员社区化管理。

　　加快建立公共财政体制。认真管好用好财政资金，合理配置公共资源。扩大推行财政支持生产资金安排的公开招投标制度，增加财政资金使用审批透明度。认真贯彻《政府采购法》，扩大政府采购范围和规模，尤其要增加工程和服务项目。扩大部门预算实施范围，实行国库集中收付制度。加强对国有资产收益、国土收入和城市经营权收益的管理，政府部门所有收入要直接缴交国库帐户。所有执收执罚的行政事业单位的预算外资金全部实行“收支两条线”管理，并实行收支脱钩的财政综合预算。各部门负责人不能自行批准减免行政事业性收费。采取有效措施，逐步提高和统一机关事业单位干部职工工资和岗位补贴。扩大向市属企业集团公司、重点基建项目和财政性资金收支较大的市直行政单位委派财务总监。成立非经营性的国有资产投资管理公司，统一管理财政性投资的项目和资产。加强税收征管，增加财政收入。充分发挥审计监督作用，强化对财政资金和国有资产的监督。

　　切实关心困难群众生活。继续采取多种形式，向广大群众征询解决生产生活困难的意见建议，满腔热情地为基层群众多办实事。年内重点工作包括：加快廉租房建设，增加廉租房有效供应，致力解决现有住房特困户的困难；安排专项资金提高重点优抚对象的生活和医疗补助，解决低保对象等困难群众的基本医疗救助；研究出台相关政策措施，认真解决困难群众打官司难、住房难、看病难、子女入学难等“四难”问题；在省政府的支持下，多方筹措资金，力争两年内全部解决糖厂欠农民蔗款；支持县(市、区)解决新增教师编制、工资和辞退民师的遗留问题；积极解决市区垃圾转运慢、住宅区噪音大、街巷路灯少和交通设施落后、秩序较乱、公共交通服务不优等群众反映强烈的问题。

　　九、强化执政为民意识，建设廉洁勤政务实高效政府

　　全市各级政府和各级领导、全体公务员要强化执政为民意识，坚持做到权为民所用，情为民所系，利为民所谋，全心全意为人民服务。

　　切实转变政府职能。继续推进政资分开、政企分开、政事分开和政社分开。强化政府规范社会管理、提供公共产品、搞好公开服务、创造公平环境的职能。改进管理方式，简化审批事项，提高行政效率和服务水平。总结经验，继续搞好机关作风测评工作，以测评活动推进机关作风整改。完善行政服务中心功能，抓好人员、职权、责任到位，规范运作，强化服务。完善办事工作日、投资代理、挂牌上岗、办事柜台化等工作制度，亲近百姓和企业，切实转变工作作风。

　　切实加强廉政建设。认真落实国务院、省政府廉政工作会议精神，进一步落实党风廉政建设责任制，抓好领导干部廉洁自律和公务员的政纪教育，清正廉洁，干净干事，规范政府公务员的从政行为。建立完善行政审批责任制和过错追究制，提高政府公信力，建立健全防范和惩治腐败的机制。推进政务公开，做到内容具体、程序规范、监督有力。各级领导干部都不允许利用职权违反规定干预和插手土地出让、工程招投标、产权交易和企业经营活动，不允许伸手向基层、企业要钱要物，坚决纠正部门和行业不正之风，狠刹奢侈浪费行为。

　　努力发扬艰苦奋斗作风。务必继续保持谦虚谨慎、不骄不躁的作风，务必继续保持艰苦奋斗的作风。各级领导干部心中要时时装着湛江百姓的福祉，深入基层体察民情，虚心倾听民众心声，设身处地感受群众疾苦，亲力亲为解决群众困难。坚决反对图表面政绩、急功近利的作风、做法，少说多干，真抓实干，贵在责任，重在落实，一个一个问题解决，一个一个困难克服，一个一个项目干好，一点一滴增强人民对湛江发展的信心。

　　各位代表！

　　湛江正面临着加快发展的良好机遇，时代召唤着我们奋进。我们要高举邓小平理论伟大旗帜，全面实践“三个代表”重要思想，在中共湛江市委的坚强领导下，紧紧依靠全市人民，坚定发展信心，团结扎实苦干，发愤进取图强，聚精会神搞建设，一心一意谋发展，努力把湛江建成现代化新兴港口工业城市，加快全面建设小康社会步伐，共同创造全市人民富裕美好的生活!
